# Size evolution of ion beam synthesized Pb nanoparticles in Al

**DOI:** 10.1186/1556-276X-9-346

**Published:** 2014-07-10

**Authors:** Huan Wang, Hongzhi Zhu

**Affiliations:** 1 Chinese Academy of Engineering Physics, P. O. Box 919-71, Mianyang 621900, People’s Republic of China

**Keywords:** Ion implantation, Pb nanoparticles, Size evolution

## Abstract

The size evolution of Pb nanoparticles (NPs) synthesized by ion implantation in an epitaxial Al film has been experimentally investigated. The average radius *R* of Pb NPs was determined as a function of implantation fluence *f*. The *R*(*f*) data were analyzed using various growth models. Our observations suggest that the size evolution of Pb NPs is controlled by the diffusion-limited growth kinetics (*R*^2^∝*f*). With increasing implantation current density, the diffusion coefficient of Pb atoms in Al is evident to be enhanced. By a comparative analysis of the *R*(*f*) data, values of the diffusion coefficient of Pb in Al were obtained.

## Background

The novel properties of embedded metallic nanoparticles (NPs) are currently the subject of intense research activities driven both by fundamental interest and by their possible applications. Among different possible techniques, high fluence implantation of an insoluble element in a crystalline matrix proved to be suitable in obtaining NP-based materials. The size control of NPs during implantation and subsequent annealing is one of the challenging issues of this approach, since the resulting thermal, optical, magnetic, and superconducting properties of NPs are drastically dependent on their size [1-7]. Therefore, a better understanding of the influence of synthesis parameters, such as implantation fluence and temperature, on average particle size during implantation is of major importance.

In this research, we have investigated the growth kinetics of embedded Pb NPs in Al during the implantation process. The ion beam synthesized Pb NPs were observed to precipitate in a crystalline Al matrix at room temperature [8]. By comparing with the theory of NP growth mechanism, a detailed description of the Pb NP nucleation and size evolution in Al is given. Finally, we obtain estimates for the following: (i) the concentration threshold for precipitation of ion beam synthesized Pb NPs in Al and (ii) the current density-dependent diffusion coefficient of Pb atoms in Al during the implantation at room temperature.

## Methods

### Epitaxial Al film deposition

Al films can be epitaxially grown on 7 × 7 reconstructed Si(111) [9]. In this work, Si(111) wafers with resistivity of 8 to 12 Ωm were used as a substrate. The Si wafers were first cleaned ex situ in a 2% hydrofluoric acid solution and subsequently in situ using a two-step silicon-flux method (silicon beam clean) [10]. This procedure results in a Si(111) surface which is free of contaminants and which exhibits the Si(111) 7 × 7 reconstruction, as confirmed by in situ reflection high energy electron diffraction and scanning tunneling microscopy. A 150-nm-thick Al layer was then evaporated at room temperature in a molecular-beam epitaxy setup with a base pressure of 5 × 10^-11^ Torr. The deposition rate (approximately 0.2 Å/s) was monitored in situ with a quartz crystal microbalance which is calibrated using X-ray reflectivity. After deposition, the sample was annealed in situ at 350°C for 2 h in order to improve the crystalline quality of Al films.

### Ion implantation

Ion implantation was performed at room temperature using Pb^+^ ions at 90 keV with implantation fluences ranging from 0.4 × 10^16^ to 1.2 × 10^17^ cm^-2^. In order to reduce the lattice damage, a channeling geometry was used [11]. The implanted sample was fixed by a clamp pressing the wafer on the sample holder, which is made of stainless steel. By tuning the anode current, the beam current extracted from ion source was controlled. The current densities were maintained at 0.5, 1.0, and 2.0 μAcm^-2^, respectively, for each sample set with a current fluctuation < 5% during implantation.

### Structural characterization

Rutherford backscattering spectrometry (RBS) with a 2.023 MeV He^+^ beam was used to determine the Pb content and Pb depth distribution in the samples, whereas the crystallinity of the Al films is assessed by ion channeling, i.e., RBS with the ion beam directed along a high-symmetry crystal direction. The minimum yield *χ*_min_, which is the ratio of backscattering yield with aligned versus random beam incidence, is a direct measure of the crystalline quality of a film [12]. The backscattered He^+^ particles were detected by two Au-Si surface barrier detectors with an energy resolution of about 15 keV, which were placed at backscattering angles of 10° and 72°, respectively.

Conventional room temperature X-ray diffraction (XRD) was performed on a Bruker D8 diffractometer using Cu K_α1_ radiation with a wavelength of 0.1542 nm. We used *θ*-2*θ* scans to identify the orientation of the epitaxial Al film and the embedded Pb NPs and to estimate the average size of the embedded Pb particles from the width of diffraction peak using the Scherrer equation [13].

## Results

### Virgin Al film on Si(111)

**Figure 1:**
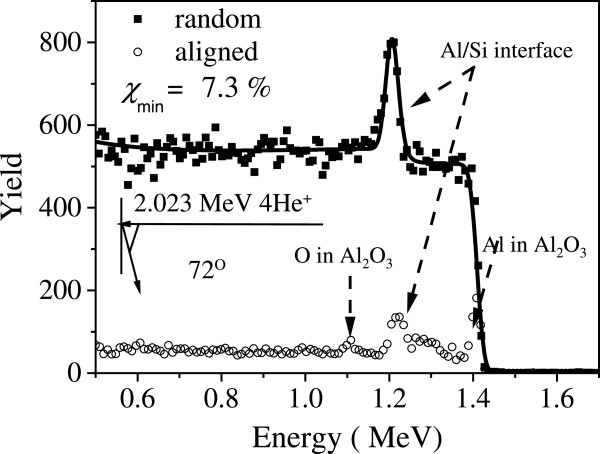
**RBS/channeling for Al/Si heterostructure.** Random (■), aligned (○), and simulated (—) spectra of 2.023 MeV He^+^ backscattered from the Al film on Si (111).

Before ion implantation, the structure of the epitaxial Al layers, which served as the matrix for embedded Pb NPs, was characterized by RBS/channeling and XRD. Figure 1 shows the random and aligned RBS spectra of the virgin Al film grown on Si(111). The detector geometry used in this backscattering measurement is shown in the inset. Because Si is heavier than Al, there is an overlap of the backscattering signals at the Al/Si interface, resulting in a peak around a backscattering energy of 1.25 MeV. In the aligned spectrum, there are two additional peaks due to the scattering from Al and O in the amorphous Al_2_O_3_ surface oxide (typically approximately 4 nm thick), which formed upon exposure of the sample to air. The low value of *χ*_min_ = 7.3% indicates a high crystalline quality of the Al film. A simulation of the random spectrum (Figure 1) by the RUMP code [14] reveals that the thickness of the Al film is 150 nm.

**Figure 2:**
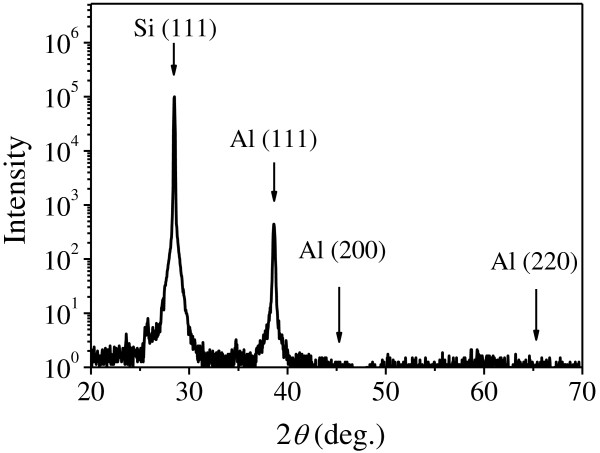
**XRD *θ*-2** ***θ *** **scans of the Al/Si heterostructure.**

The symmetric XRD *θ*-2*θ* scans of the Al/Si(111) heterostructure in the 2*θ* range 20° to 70° are shown in Figure 2. The only Al peak that can be detected is the Al(111) diffraction peak at 2*θ* ≈ 38.5°, illustrating that the crystalline Al film is highly oriented with respect to the Si substrate as Al(111)//Si(111).

### Determination of the implanted Pb content and depth distribution

**Figure 3:**
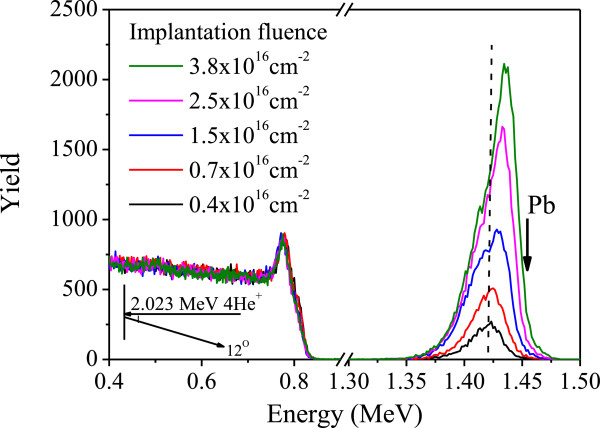
**Random RBS spectra for the samples with fluences ranging from 0.4 × 10**^**16**^**to 3.4 × 10**^**16**^ **cm**^**-2**^**.** Implantation current density is 2.0 μAcm^-2^. The dashed line is a guide for the eye for the shift of the depth profile with increasing fluence. The arrow labeled with Pb indicates the energy for backscattering from Pb atoms at the surface.

Immediately after implantation, the implanted Pb content and Pb depth profile in Al were obtained from the experimental RBS spectra. Figure 3 shows the random RBS spectra of the samples with the same implantation current density at 2.0 μAcm^-2^ but different implantation fluences (<4.0 × 10^16^ cm^-2^). The detector geometry is shown in the inset. At low fluences, Pb is deposited inside the Al layer and only Al can be sputtered. This leads to a recession of the surface and a shifting of the Pb peak to the sample surface. After careful analysis of the RBS spectra, an average experimental sputtering yield is estimated to be approximately 3.2, which is smaller than the result of Stopping and Ranges of Ions in Matter (SRIM) simulation (7.0 ± 0.2) for random implantation in pure Al [15]. The reduced sputtering yield is probably due to the lower deposited energy density at the surface for the channeled ions compared to the random implanted ions [16]. Our results show that the sputtering yield of channeled Pb implantation is reduced by a factor 2.2 compared to the one of non-channeling implantation (obtained from SRIM simulation). This reduction is consistent with a reduction by a factor of 2 to 5, which is generally found for bombardment close to the major crystal axes with respect to other directions in single-crystalline targets [17]. In addition, with increasing fluence, the increased stopping power (both elastic and inelastic) in the Pb-enriched zone results in a reduced projected range of implanted Pb ions. The fluence-dependent projected range not only causes the Pb depth profile to move towards the surface but also leads to an enhancement of Pb concentration in the Pb-enriched zone. When the Pb depth profile reaches the surface, Pb starts to get self-sputtered. In this case, if the sputtering yield of Pb is larger than 1, a decrease of the Pb content with increasing implantation fluence can be observed. In our research, a significant decrease of Pb content is found for the samples with the implantation fluence of 6.0 × 10^16^ cm^-2^. Such phenomenon has also been observed in implanted Si systems and explained well by Eckstein [18,19]. For higher implantation fluences, the Pb content saturates at 2.7 × 10^16^ cm^-2^ indicating that a steady state is reached between the ions removed by surface sputtering and those added via implantation. By assuming the sputtering yield of Al is the same as the one with low implantation fluence (<4.0 × 10^16^ cm^-2^), the sputtered thickness of Al at the beginning of the steady state (with the fluence of 8.0 × 10^16^ cm^-2^) is estimated to be approximately 41 nm, which is comparable with the projected range of 90 keV Pb in Al (36 nm).

**Figure 4:**
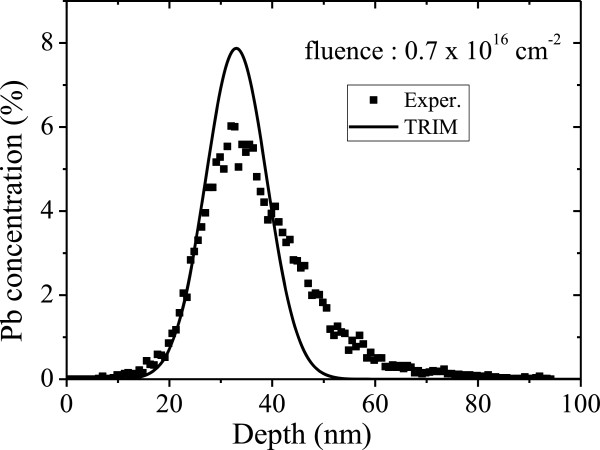
**Experimental Pb depth profile in Al (solid squares) obtained from RBS.** The solid line is a theoretical profile obtained from the TRIM program.

The Pb depth profile for the sample with the implantation fluence *f* = 0.7 × 10^16^ cm^-2^ is shown in Figure 4. Compared with the simulated depth profile obtained from the Transport of Ions in Matter (TRIM) program (with a random incident ion implantation) [20], the broadening of the Pb depth profile obtained from RBS result is much larger. This can be attributed to (i) the relatively lower stopping power for channeling implanted ions and (ii) migration of Pb atoms in Al caused by the ion irradiation related heating effects [21].

### Size evaluation of Pb nanoparticles in Al

**Figure 5:**
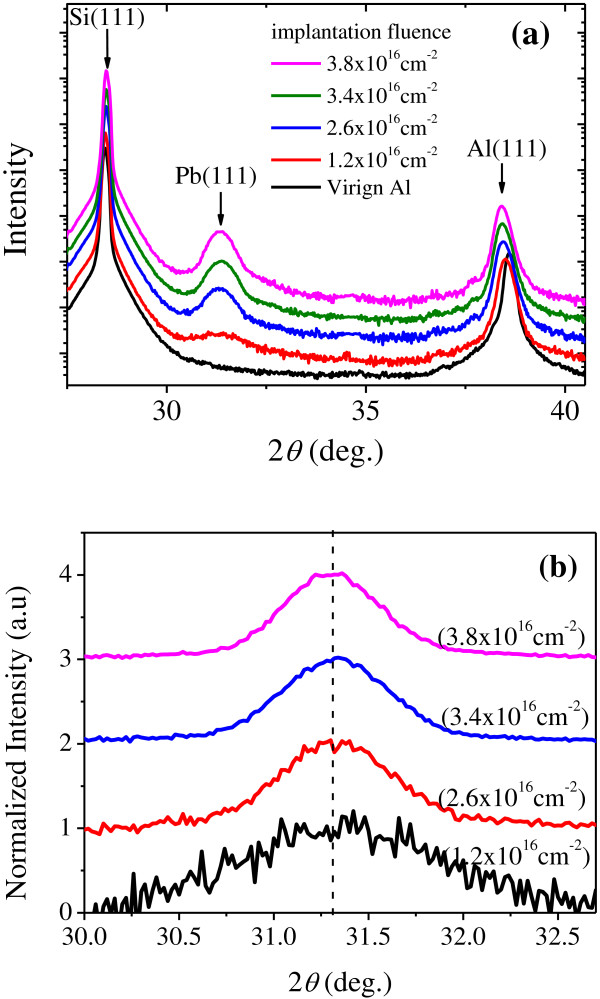
**XRD *θ*-2*θ *scans (a) and (b) for the samples with different implantation fluences.** The implantation current density is 2.0 μAcm^-2^. The arrows in (a) show the positions of Si(111), Pb(111), and Al(111) diffractions, respectively. The dashed line in (b)indicates the peak position of bulk Pb(111) diffractions. The diffraction profiles are shifted vertically for clarity.

Figure 5a shows the XRD *θ*-2*θ* scans for a virgin Al sample and for the samples with the implantation current density at 2.0 μAcm^-2^ and implanted up to different fluences. For all samples, the only detectable Pb peak is the Pb(111) diffraction at 2*θ* ≈ 31.3°, confirming that the Pb particles are highly oriented with respect to the host Al(111) matrix [8]. The defects, such as vacancies, introduced by ion bombardment are expected to lead to a peak shift of Al. Such phenomenon is generally observed in implanted systems [22]. In order to accurately determine the lattice of the Pb NPs, XRD signals from the Pb NPs were carefully monitored by *θ*-2*θ* scans with 2*θ* ranging between 30.0° and 32.7°. The Pb(111) diffraction profiles of the samples with different implantation fluences are plotted in Figure 5b after subtracting the background signal. It can be seen that all the peak positions are consistent with the bulk value (31.30°) indicating that the embedded Pb NPs are strain free. The commensurate condition 4*a*_Pb_ ≈ 5*a*_Al_, with *a*_Pb_ and *a*_Al_ the lattice parameters of Pb and Al, indicates a small lattice mismatch within 2% [23]. As such, the strain can be expected to be easily released by misfit dislocations at interfaces.

**Figure 6:**
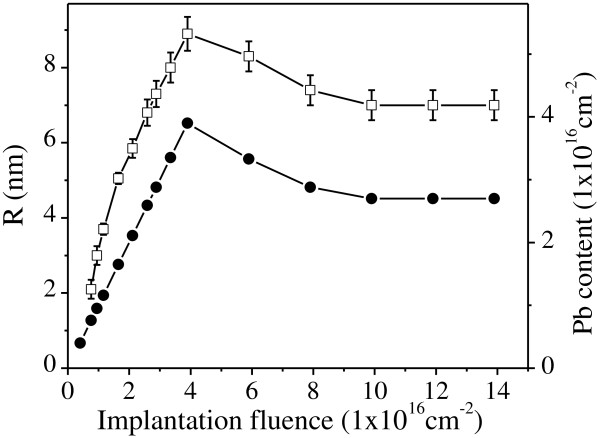
**Pb content (●) and average radius (□) of the Pb NPs versus implantation fluence *f*.**

It is well known that Bragg peaks are broadened as the coherent diffracting region becomes spatially smaller. The average size of the diffracting region (*d*) can be approximately related to the full width at half maximum *B* of a Bragg peak in a 2*θ* scale through the Scherrer formula [13]:

(1) $$d=\frac{\mathrm{K\lambda}}{B\cos\theta }$$

where *λ* is the X-ray wavelength, *θ* is the Bragg angle, and *K* is a constant of the order of unity whose exact value depends on the specific shape and crystallographic direction of the diffracting planes [13]. Calculated *K* values for the (111) direction in many different shapes and structures are close to 0.9 to within a few percent [13], so we have consistently adopted this value for the Pb(111) reflection. Assuming a spherical shape, the average radius (*R* = *d/*2) of the Pb NPs can then be deduced from the XRD patterns, which is shown in Figure 6 by the squares. It can be seen that the average radius of the Pb NPs scales with the implanted Pb content up to a maximum of 8.9 nm and subsequently saturates at about 7.2 nm.

## Discussion

### Theoretical background

In order to explain the size evolution of the Pb NPs under our experimental conditions, the classical nucleation and growth theory which has been developed for ion implanted systems can be used [24-26]. The formation and growth of NPs during ion implantation can be divided into three distinct stages:

#### *Supersaturation*

At the early stage of implantation, the impurity atoms are found as dissolved monomers. Depending mainly on the mobility of the implanted atoms, they can either remain ‘frozen’ in their final position or may subsequently diffuse through the lattice. During implantation, the concentration of monomers *C*_*m*_ increases linearly with time. Since ion implantation is not a thermodynamic equilibrium process, the solubility limit of the implanted ions in the host can be largely exceeded, achieving impurity concentrations higher than the bulk solubility, *C*_∞_.

#### *Nucleation*

In the case of non-zero mobility, as *C*_*m*_ increases further and exceeds a critical value *C*_*C*_, small agglomerates of impurity atoms (i.e., dimers and trimers) start to form. Consequently, the increase of *C*_*m*_ slows down. Subsequently, these tiny agglomerates constitute a pool of nucleation sites and some of them grow (by statistical fluctuations) beyond a critical radius *R*_C_, thus forming stable precipitates. Here, *R*_C_ represents the critical radius above which a particle spontaneously grows and below which it dissolves. These stable precipitates act as sinks for diffusing monomers. Despite the fact that the impurity atoms are continuously implanted, *C*_*m*_ starts to decrease and eventually drops below the concentration threshold *C*_*C*_.

#### *Growth*

As soon as *C*_*m*_ drops below *C*_*C*_, no new particles are formed and the existing ones grow by incorporation of newly implanted impurity atoms. The growth of NPs is driven by the transport of the monomers to the particle/matrix interface, i.e., by diffusion, and then by their absorption and incorporation into the particle via interface interactions. The growth rate *dR*/*dt* of a spherical particle of radius *R*(*t*) can be thus described by a general expression, which includes both diffusion and interface absorption [26-29]:

(2) $$\frac{\mathrm{dR}}{\mathrm{dt}}=k\left(C_{m}-C_{\infty }\right)\left(\frac{1-R_{C}/R}{1+\mathrm{\epsilon R}}\right)$$

where *k* is the rate of monomer absorption at the particle surface, *ϵ*^-1^ = *DV*_*a*_/*k* is the screening length which compares bulk diffusion to surface integration effect, *D* is the diffusion coefficient of Pb atoms in Al, and *V*_*a*_ is the molar volume of Pb precipitates.

To retrieve the particle growth law in the growth regime, we assume *R* ≫ *R*_*C*_. The product *ϵR* = *kR*/*DV*_*a*_ is the key parameter determining the growth mechanism. When *kR* ≪ *DV*_*a*_, the interface integration is the rate-determining step. In this case, integration of Eq. (2) reveals that the particle size increases linearly with time during the growth regime, i.e., *R*∝*t*, with a slope of *k*(*C*_*m*_ - *C*_∞_). On the other hand, when *kR* ≫ *DV*_*a*_, the growth is purely diffusion limited and presents different kinetic behavior as *R*^2^∝*t* with a slope of 2*DV*_*a*_(*C*_*m*_ - *C*_∞_). While, if *kR* is comparable with *DV*_*a*_, the growth rate is determined by both diffusion and interface absorption, the precipitates evolve as (*ϵR*^2^ + *2R*) ∝*t*. For ion implantation with a constant current density since implantation fluence *f*∝*t*, it can be seen that the scaling law of the average particle radius *R* with implantation fluence *f* provides a distinct signature for distinguishing the growth kinetics of the embedded NPs. In addition, the important values of the absorption rate *k* (in the interface kinetic limited case) and the diffusion coefficient *D* (in the diffusion limited case) during implantation can be deduced.

### Size evolution of Pb nanoparticles

Due to the extremely small value of *C*_∞_ for Pb in Al (0.19 at.% at 601 K) [30], the supersaturation and nucleation regimes should already be finished after a short implantation time, i.e., at a low implantation fluence. It was observed that Pb NPs with average radius about 2.1 nm are formed with an implantation fluence of 7 × 10^15^ cm^-2^ and a current density at 2.0 μAcm^-2^ (Figure 6). Thus, the upper limit of the critical monomer concentration for particle nucleation to occur *C*_*C*_ can be estimated to be 6 at.% in Al, i.e., 6.2 × 10^-3^ mol/cm^3^, by assuming that all the implanted Pb atoms (7 × 10^15^ cm^-2^) are dissolved monomers in the Al layer (Figure 4). In addition, since *C*_*m*_ < *C*_*C*_ in the growth regime, one can safely assume the upper limit of *C*_*m*_ = *C*_*C*_ = 6.2 × 10^-3^ mol/cm^3^ during the implantation process.

**Figure 7:**
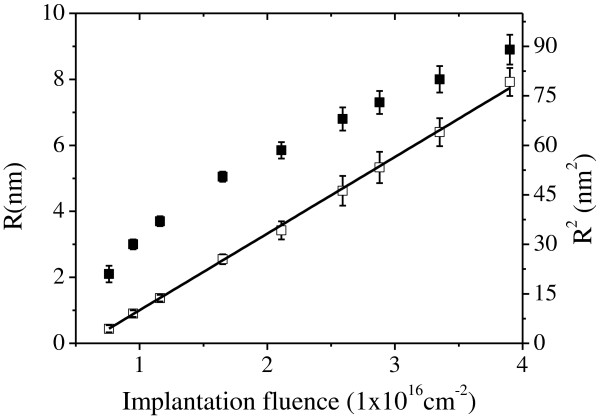
***R *(■) and *R***^**2 **^**(□) versus implantation fluence.** The solid line (—) is the diffusion growth model fitted to the experimental data.

To better demonstrate the size evolution of embedded Pb particles after supersaturation and nucleation regimes, we report in Figure 7 both *R* and *R*^2^ of the growing particles as a function of implantation fluence *f*. There is a linear relation between *R*^2^ and *f*, indicating the diffusion limited growth of embedded Pb NPs with their average radius ranging from 2.1 to 8.9 nm. Moreover, the lower limit of diffusion coefficient *D* = 0.15 nm^2^/s is obtained by neglecting *C*_∞_ and assuming the molar volume of Pb precipitates *V*_*a*_ to be that of bulk Pb and the upper limit of *C*_*m*_ to be that of *C*_*C*_. The motion of Pb atoms is expected to be assisted by the radiation induced collision cascade and vacancies. When the implantation fluence exceeds 4.0 × 10^16^ cm^-2^, the Pb NPs exposed at the sample surface start to be sputtered.

**Figure 8:**
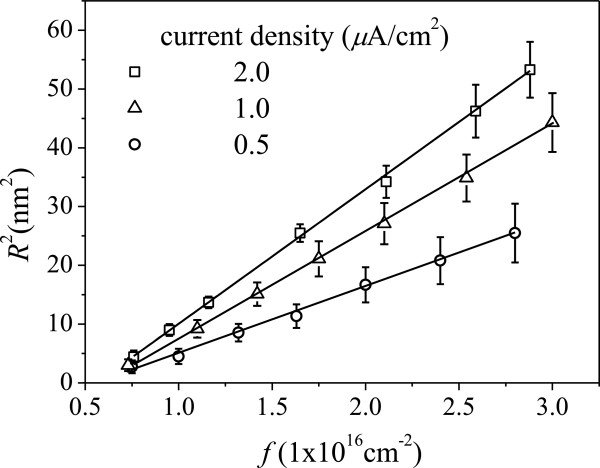
***R***^**2 **^**versus implantation fluence with different implantation current densities.** The solid line (—) is the diffusion growth model fitted to the experimental data.

The aggregation of Pb into NPs in these implanted samples occurs even after room temperature implantation with no further annealing suggesting a high mobility of implanted Pb atoms in Al and some beam heating effects were present. To study the dynamic effects involved, we examined the current density dependence of the size evolution of Pb NPs. Figure 8 shows the *R*^2^ of the growing particles as a function of implantation fluence *f* with different implantation current densities. A linear relation between *R*^2^ and *f* with a changed slope is identified by changing the implantation current density *φ* from 0.5 to 2.0 μA/cm^2^. The variation of slope in the plot of *R*^2^ versus *f* suggests a change of the diffusion coefficient *D* of Pb atoms in Al, which is estimated to be 0.15, 0.08, and 0.04 nm^2^/s, respectively, by decreasing current density. The dependence clearly demonstrates that the aggregation process of the implanted Pb is altered by a change in ion-beam current density. During implantation, the sample was heated caused by the beam bombardment. In previous investigations, significant temperature enhancement, which is current density dependent, was observed in implanted samples [31,32]. In our case, the closed contact between the sample and its holder is expected to reduce the heating effect compared to the case with limited contact. However, the residual heat in sample is still evident to be current dependent and to increase the temperature of the samples allowing enhanced migration, i.e., high diffusion coefficient, of Pb atoms and thus coalescence into larger Pb NPs.

## Conclusions

We have investigated the clustering process of Pb atoms implanted in a single crystalline Al layer grown on Si(111). By analyzing the average particle radius *R* as a function of implantation fluence *f*, we observed the diffusion limited growth of ion beam synthesized Pb NPs during the implantation process. Moreover, with a decreasing implantation current density from 2.0 to 0.5 μAcm^-2^, a lower limit of the diffusivity of Pb in Al ranging from 0.15 to 0.04 nm^2^/s was obtained. This phenomenon indicates that implantation current density is one of the parameters which can be applied to tune the particle size during the implantation process.

## Competing interests

The authors declare that they have no competing interest.

## Authors' contributions

HW designed the experiments and wrote the manuscript. HZ supervised the whole work. Both authors read and approved the final manuscript.
